# Precision Photothermal Therapy at Mild Temperature: NIR‐II Imaging‐Guided, H_2_O_2_‐Responsive Stealth Nanobomb

**DOI:** 10.1002/adhm.202402767

**Published:** 2024-10-10

**Authors:** Gongcheng Ma, Qihang Ding, Yue Wang, Zhiwei Zhang, Yuding Zhang, Hui Shi, Lintao Cai, Ping Gong, Pengfei Zhang, Zhen Cheng, Jong Seung Kim

**Affiliations:** ^1^ School of Life Science and Technology Xinxiang Medical University Xinxiang 453003 P. R. China; ^2^ Department of Chemistry Korea University Seoul 02841 South Korea; ^3^ Guangdong Key Laboratory of Nanomedicine CAS‐HK Joint Lab of Biomaterials CAS Key Laboratory of Biomedical Imaging Science and System Shenzhen Engineering Laboratory of Nanomedicine and Nanoformulations CAS Key Lab for Health Informatics Shenzhen Institutes of Advanced Technology Chinese Academy of Sciences Shenzhen 518055 P. R. China; ^4^ University of Chinese Academy of Sciences Beijing 100049 P.R. China; ^5^ State Key Laboratory of Drug Research Molecular Imaging Center Shanghai Institute of Materia Medica Chinese Academy of Sciences Shanghai 201203 P. R. China; ^6^ Sino‐Euro Center of Biomedicine and Health Luohu Shenzhen 518024 P. R. China

**Keywords:** aggregation‐induced emission, carbon monoxide, heat shock proteins, mild‐temperature photothermal therapy, NIR‐II imaging

## Abstract

The therapeutic efficacy of photothermal therapy (PTT) under mild temperatures (<45 °C) is hindered as cancer cells can activate heat shock proteins (HSPs) to mend fever‐type cellular damage swiftly. The previous attempt fabricated first‐generation nanobombs (nanobomb1G) by self‐assembly of polymeric NIR‐II AIEgens and carbon monoxide (CO) carrier polymer mPEG(CO) to inhibit the expression of HSPs after intratumor injection. A new generation nanobomb (Stealth NanoBomb (SNB)) is developed by self‐assembling small molecular NIR‐II AIEgens with CO carrier polymer PLGA(CO) coated by PEG‐lipid. This design allows for intravenous administration, enabling the SNB to circulate safely in the bloodstream and selectively target cancer cells. Upon accumulation in tumors, the SNB releases CO to effectively suppress HSP expression, enhancing the therapeutic efficacy of mild‐temperature PTT. Compared to the previous generation, the SNB offers a safer, more stable, and more efficient CO gas/drug co‐delivery system for cancer treatment. This work represents a significant advancement in PTT, providing a promising strategy for enhanced antitumor therapy with reduced systemic toxicity.

## Graphical abstract

The new generation nanobomb, termed the stealth nanobomb, is fabricated through self‐assembly using a polymeric carbon monoxide carrier (PLGA(CO)), small molecule near‐infrared‐active agents (2TT‐OC46B), and phospholipid polyethylene glycol (DSPE‐mPEG2000). The stealth nanobomb can circulate in the bloodstream and specifically target pancreatic cancer, enabling mild temperature photothermal therapy.

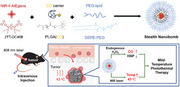

## 1 Introduction

Photothermal therapy (PTT), a localized and noninvasive therapeutic approach for treating solid tumors, has attracted considerable interest in the biomedical field.^[^ ^1^, ^2^ ^]^ However, achieving complete tumor elimination requires raising the temperature of cancerous tumors to over 50 °C.^[^ ^3^ ^]^ Such elevated temperatures can target and ablate tumors but inadvertently damage surrounding normal tissues due to heat diffusion.^[^ ^4^, ^5^ ^]^ The therapeutic efficacy of PTT under mild temperature (<45 °C) is hindered as cancer cells can initiate self‐preservation mechanisms, like heat shock proteins (HSPs), to mend fever‐type cellular damage swiftly.^[^ ^6^, ^7^ ^]^ To bolster the therapeutic effects of PTT, various strategies have been adopted to inhibit HSPs, including inhibitors, siRNAs, cytokine, single atom nanozyme, etc.^[^ ^8^, ^9^, ^10^, ^11^ ^]^ In our previous work, we engineered a nanobomb through the self‐assembly of polymeric NIR‐II AIEgens and PEG‐pentacarbonyliron conjugate, in which carbon monoxide (CO) could serve as gaseous inhibitors for inhibiting the upregulation of HSPs during PTT process under mild temperature.^[^ ^12^ ^]^ Unfortunately, the nanobomb could only be developed based on polymeric NIR‐active agents. Besides, the loose structure of nanobomb posed a potential risk of CO leakage in the blood, which led to the administration of nanobomb being restricted to intratumoral injection.

Since 2008, the lipid‐polymer hybrid nanoparticle (NP) has served as a stealth drug delivery platform, offering high drug encapsulation yield, a tunable and sustained drug release profile, exceptional serum stability, and the potential for targeted delivery to specific cells or tissues.^[^ ^13^ ^]^ In this system, Poly (D, L‐lactide‐co‐glycolide) (PLGA) was employed as an inner core material, which was further coated with polyethylene glycol decorated lipid (PEG‐lipid), providing a stealth delivery platform to evade the immune system, thereby prolonging their circulation time and improving the efficiency of drug delivery.^[^ ^14^, ^15^, ^16^, ^17^ ^]^ In prior studies, these PEG‐shell lipid‐polymer hybrid NPs have found extensive application in delivering diverse therapeutic agents such as drugs, genes, protein vaccines, peptides, diagnostic imaging agents, and targeting ligands, offering a new avenue for the fabrication of safer CO gas delivery systems.^[^ ^18^, ^19^, ^20^ ^]^

**Scheme 1:**
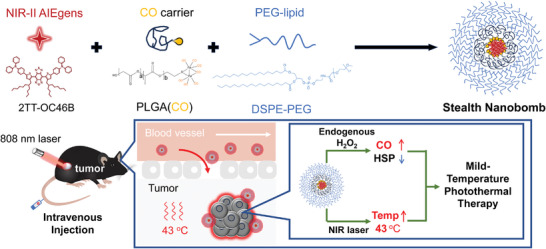
Schematic illustration of **SNB** and its application in the mild temperature PTT process.

In this work, the new generation nanobomb (termed Stealth NanoBomb (**SNB**)) was fabricated through self‐assembling using polymeric CO carrier (PLGA(CO)), small molecule NIR‐active agents (2TT‐OC46B), and phospholipid polyethylene glycol (DSPE‐mPEG2000). The **SNB** could circulate in the blood and target pancreatic cancer (PC) for mild temperature PTT (**Scheme** **1**).

## 2 Results and Discussion

**Figure 1:**
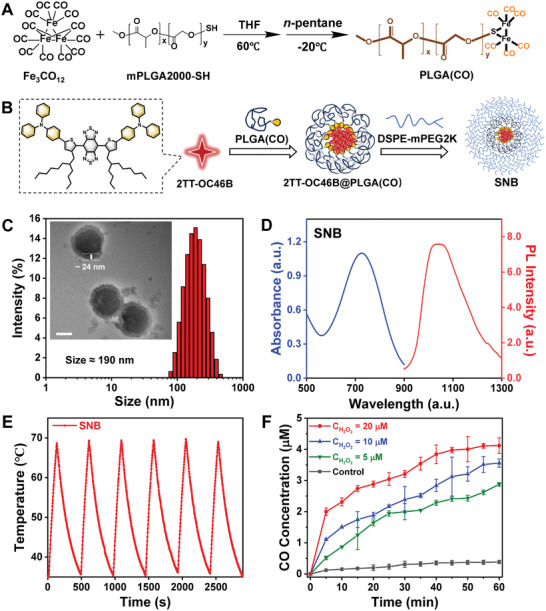
Synthesis diagram and characterization of **SNB**. A) Schematic diagram of PLGA(CO) synthesis route. B) Schematic diagram of the preparation of **SNB**. C) The TEM image and DLS size of **SNB**. Scale bar 100 nm. D) The absorbance and fluorescence spectra of **SNB**. E) In six cycles, photothermal heating and cooling curves of **SNB** under 808 nm laser. F) The release rate of CO with the concentration changes of H_2_O_2_.

The small molecule NIR‐active agent 2TT‐OC46B was synthesized according to previous work with a little modification. (Figure S1–S3, Supporting Information) 2TT‐OC46B showed near‐infrared absorbance and typical aggregation‐induced emission (AIE) characteristics, with fluorescence emission ranging from 900–1200 nm (Figure S4, Supporting Information). As depicted in **Figure** **1**A, PLGA(CO), as the CO storage polymer, was synthesized from dodecarbonyltriiron and mercaptopoly(lactic‐co‐glycolic acid) (PLGA2000‐SH). This polymer was then self‐assembled with 2TT‐OC46B to produce a chemiexcitation‐triggered AIE NP named 2TT‐OC46B@PLGA(CO). Subsequently, 2TT‐OC46B@PLGA(CO) was co‐precipitated with phospholipid polyethylene glycol (DSPE‐mPEG2000) to create a **SNB** featuring a core‐shell structure (Figure 1B). As prepared, the 2TT‐OC46B@PLGA(CO) exhibited a spherical morphology and core structure characterized by sizes of ≈151.5 nm, as observed by transmission electron microscopy (TEM). (Figure 1C). By contrast, a high‐resolution TEM image confirms the successful preparation of the core‐shell nanostructure of **SNB** with a size of ≈175.5 nm; the average shell thickness was measured to be 24 ± 0.6 nm. The dynamic light scattering (DLS) analysis indicated that both spherical 2TT‐OC46B@PLGA(CO) and **SNB** possessed average sizes of ≈164 and 190 nm, respectively, consistent with the TEM results (Figures S5 and S6, Supporting Information). The element distribution diagram illustrates the homogeneous dispersion of N, O, S, and Fe, providing additional confirmation of the formation of 2TT‐OC46B@PLGA(CO) and **SNB** (Figures S7 and S8, Supporting Information). Besides, the surface zeta potential changed from −24 ± 0.9 to −18.0 ± 0.3 mV (Figure S9, Supporting Information) due to charge screening by the DSPE‐mPEG. Upon preparation, **SNB** exhibited broad absorption ranging from 580 to 810 nm, peaking at ≈730 nm (Figure 1D; S10, Supporting Information). When excited by an 808 nm laser, 2TT‐OC46B@PLGA(CO) displayed robust fluorescence in the NIR‐II region, centered at 960 nm. Meanwhile, **SNB** showed a red‐shifted absorption peak at 1035, which is suitable for NIR‐II imaging. As shown in Figure 1E and Figure S11–S13 (Supporting Information), **SNB** can effectively convert NIR light energy into heat with photothermal conversion efficiency (η) as high as 33.1%. The **SNB** exhibits a fluorescence quantum yield of ≈12.68% in in aqueous solution. Moreover, there was negligible deterioration of the photothermal effect after six cycles of heating and cooling processes, reflecting the photostability of **SNB** (Figures S14 and S15, Supporting Information).

Moreover, the in vitro capacity of **SNB** to release CO was confirmed, as illustrated in Scheme S1 (Supporting Information). Reduced hemoglobin (Hb) served as the metric for quantifying the liberated CO content. As depicted in Figure 1F, the CO content was determined based on the absorption peak value of reduced Hb and HbCO. Previous studies have indicated that the concentration of H_2_O_2_ generated by an individual tumor cell is ≈10 µm. To evaluate both the quantity and rate of CO release, experiments were conducted over a 1‐h period at different H_2_O_2_ concentrations (0, 5, 10, and 20 µm), replicating conditions within the tumor microenvironment and verifying the efficacy of **SNB**. In addition, Figure S16 (Supporting Information) illustrates minimal CO release when H_2_O_2_ is absent. As the concentration of H_2_O_2_ approached the expression level in tumor cells, the **SNB** released CO violently at the rate of 2008 s^−1^.

**Figure 2:**
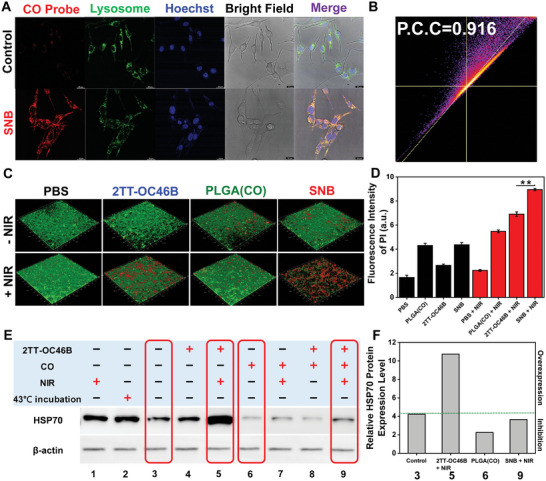
Experimental study of **SNB** at the cellular level. A) The expression of CO in Panc02 cells was imaged by the confocal fluorescence microscope. B) Intensity scatter plot of lysosome and CO probe channels. C) The viability of Panc02 cells was assessed using a 3D confocal fluorescence microscope in the live–dead staining experiment. D) Statistics of fluorescence signal intensity expressed by PI. (^*^ *p* < 0.05, ^**^ *p* < 0.005, and ^***^ *p* < 0.0005). E) Western blot images of HSP70 with different treatments. F) Corresponding gray analysis of HSP70 with different treatments.

Termed the “king of cancer,” PC stands as a leading cause of cancer‐related deaths worldwide, made especially challenging by its unique tumor microenvironment characterized by a high hydrogen peroxide (H_2_O_2_) microenvironment.^[^ ^21^, ^22^ ^]^ The controlled release process in PC cells (Panc02 cells) and normal cells (LO2 cells and RAW264.7 cells) was evaluated through confocal laser scanning microscopic (CLSM) imaging. The intracellular CO release was assessed employing a CO probe, manifesting alterations in absorption at 550 nm and an induced fluorescence turn‐on signal at ≈570 nm. As demonstrated in **Figure** **2**A and Figures S17–S20 (Supporting Information), robust fluorescent signals from the CO probe were observed exclusively in Panc02 cells after incubation with SNB, instead of normal cells, immune cells, and oxidative stress processes caused by drug damage. This indicates that SNB can selectively release CO gas in the Panc02 tumor microenvironment, which has high levels of H_2_O_2_. Moreover, the confocal imaging experiments involving the co‐incubation of green fluorescent lysosome tracker with Panc02 cells indicated the potential localization of **SNB** within lysosomes at the cellular level. This deduction is further supported by analyzing the intensity scatter plot for the red‐green channels (Figure 2B).

Next, we investigated whether CO could enhance the PTT effect in PC. Live/dead staining was performed to evaluate the synergistic antitumor ability further. Figures 2C and S21 (Supporting Information) display the corresponding fluorescent photographs of biofilm using 3D CLSM. Panc02 cells treated with SNB were effectively inhibited under mild temperature PTT. However, individual CO or the PTA alone exhibited limited ability to kill Panc02 cells. The comparison of fluorescence signal intensity of PI can further prove the conclusion (Figures 2D; S22, Supporting Information). In addition, results from the Standard Cell Counting Kit‐8 (CCK‐8) indicated a notable reduction in the survival rate of cancer cells (Pan02) treated with **SNB** compared to normal cells (LO2) and immune cells (RAW264.7). As shown in Figures S23–S25 (Supporting Information), this observation suggests that **SNB** exhibits a selective release of CO in the tumor microenvironment characterized by elevated levels of H_2_O_2_, aligning with the findings from live/dead cell stain results.

Further, for additional confirmation of the suppressive impact of CO on HSPs expression, we conducted a western blotting to evaluate the levels of HSP70 in cells following various therapy protocols. In Figures 2E,F, and S26 (Supporting Information), the presence of CO significantly inhibits the expression of HSP70 in Panc02 cells. The upregulation of HSP70 in Panc02 cells under thermal conditions was noticeably modulated upon treatment with **SNB**. In the cancerous microenvironment, the gradually released CO could effectively inhibit the elevated expression of the HSPs to destroy tumor thermal resistance during the mild temperature PTT process and induce tumor apoptosis.^[^ ^23^, ^24^, ^25^ ^]^

**Figure 3:**
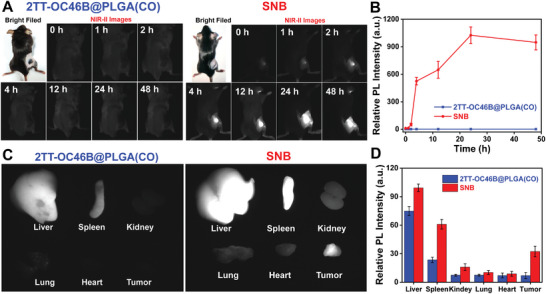
The tumor‐targeting ability of **SNB** injected intravenously. A) The NIR‐II fluorescence images of panc02 tumor‐bearing mice after intravenous injection of 2TT‐OC46B@PLGA(CO) (left) and **SNB** (right). B) The fluorescence intensity curve of the tumor within 48 h after intravenous injection of 2TT‐OC46B@PLGA(CO) (left) and **SNB** (right). C) The NIR‐II images and D) relative fluorescence intensity of heart, liver, spleen, lung, kidney, and tumor of mice after intravenous injection of **SNB** in 48 h. (^*^ *p* < 0.05, ^**^ *p* < 0.005, and ^***^ *p* < 0.0005).

Intravenous injection is one of the primary methods of drug administration to get therapeutic agents into the body. As a proof of concept, **SNB** was put into the mice bearing Panc02 tumors through intravenous injection. As shown in **Figures** **3**A and S27 (Supporting Information), **SNB** can effectively enrich the tumor site and reach its highest content at ≈24 h. Significant fluorescence at tumor sites can be gradually observed by quantifying the signal intensity at different time points, indicating that **SNB** has effectively accumulated in the tumor after intravenous administration (Figures 3B; Figures S28 and S29, Supporting Information). Furthermore, the main organs gained included the heart, liver, spleen, lung, kidney, and tumor. The organ distribution analysis indicated that **SNB** is mainly located in the liver, spleen, and tumor, consistent with previous work.^[^ ^26^, ^27^, ^28^, ^29^ ^]^ (Figure 3C; Figures S30 and S31, Supporting Information).

**Figure 4:**
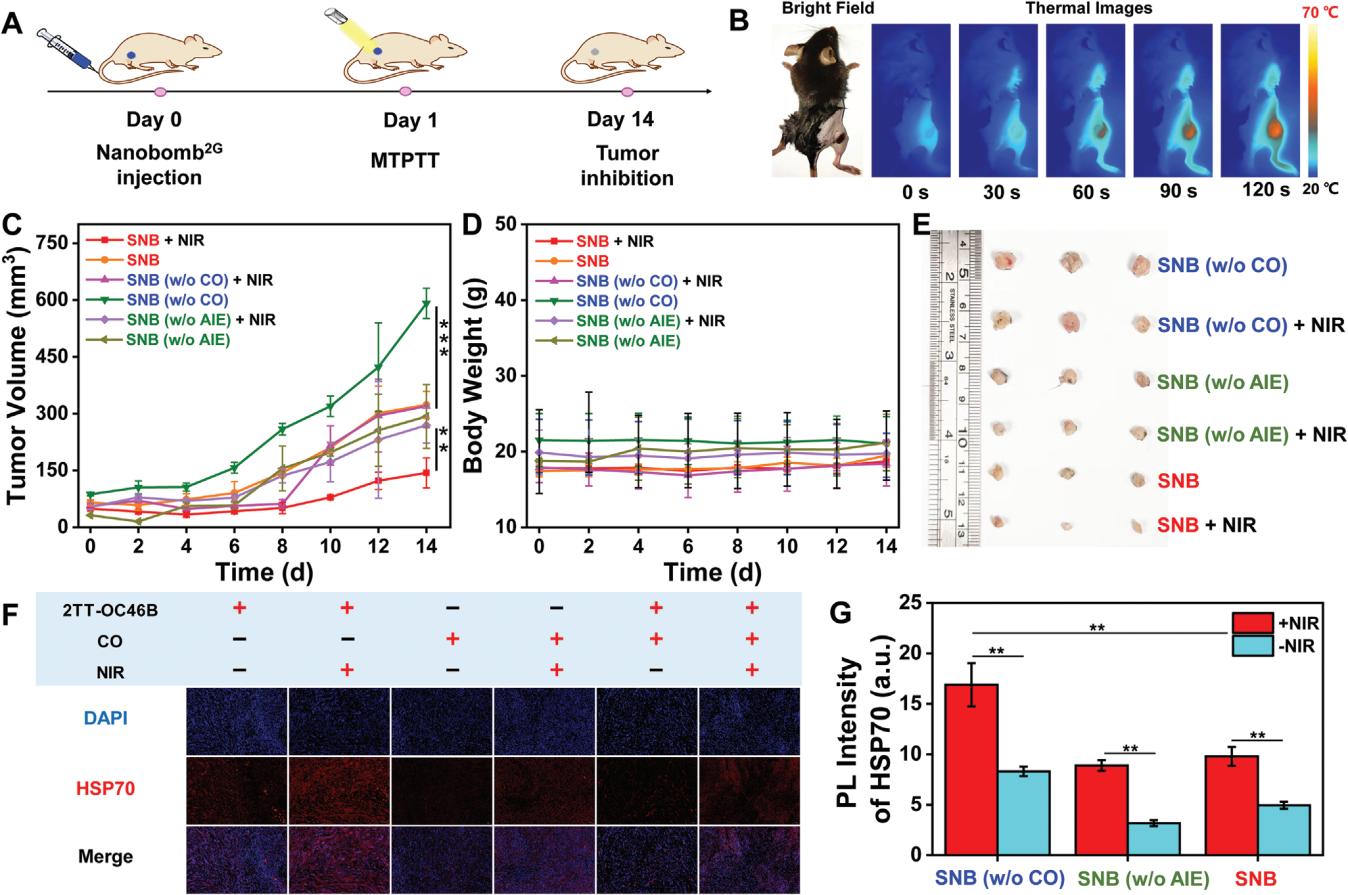
Experimental study of low‐temperature PTT with **SNB** on tumor‐bearing mice. A) Schematic diagram of the mild temperature PTT process. B) Thermal images of tumor irradiated by 808 nm laser with time following intravenous administration of **SNB**. C) Temporal evolution of mouse tumor volume over 14 days. D) Alterations in mouse body weight throughout the experiment. E) Photographs depicting the excised tumor post‐treatment. F) Staining sections of tumor tissues revealed the HSP70 expression following various treatment conditions. Scale bar 100 µm. G) The fluorescence intensity exhibited notable variations across distinct conditions. (^*^ *p* < 0.05, ^**^ *p* < 0.005, and ^***^ *p* < 0.0005).

Encouraged by the excellent tumor enrichment efficiency in vivo, we further evaluated the PC PTT effect of **SNB** in vivo. The in vivo mild temperature PTT efficiency of **SNB** was validated using a Panc02 tumor‐bearing mice model. The mild temperature PTT procedure in the Panc02 tumor‐bearing mice model is illustrated in **Figure** **4**A, where the tumor was exposed to an 808 nm laser following the intravenous administration of **SNB**. The therapeutic temperature was maintained at ≈43 °C and monitored in real‐time using a thermal images approach (Figure 4B). In contrast to the control groups, the combination therapy involving **SNB**/mild temperature PTT demonstrated effective tumor inhibition, resulting in a persistent reduction in tumor volume (Figure 4C; Figure S32, Supporting Information). The experimental mice did not show notable weight loss or abnormalities in their body weight, suggesting the absence of significant toxicity induced by **SNB**. (Figure 4D; Figure S33, Supporting Information). Ultimately, we excised and measured the size of the isolated tumor tissue in vitro. The outcomes are depicted in Figure 4E, aligning with the findings in Figures 4C and Figure S34 (Supporting Information). Furthermore, examination of hematoxylin and eosin (H&E) stained tumor sections, as illustrated in Figure S35 (Supporting Information), revealed serious destruction to tumor cells in the group of mild temperature PTT with **SNB**. In contrast, cancer cells in other groups exhibited negligible impact or limited destruction.

**Figure 5:**
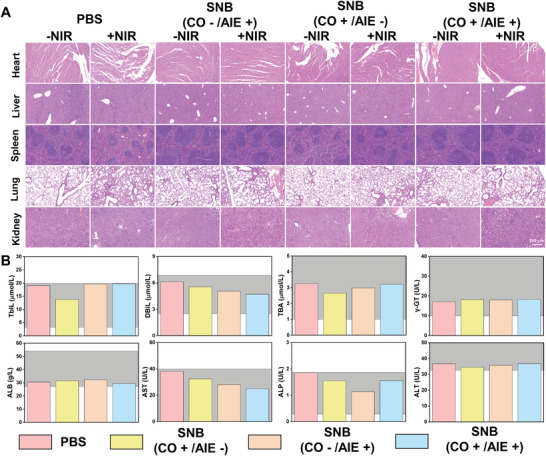
Biological safety validation of **SNB**. A) The histological analysis of the heart, liver, spleen, lung, and kidney by the H&E staining, Scale bar 200 µm. B) Blood biochemical analysis post‐injection. From left to right: PBS, PLGA(CO), 2TTOC46B, **SNB**. The gray areas indicate the reference ranges for the corresponding hematological data in standard Balb/c mice.

To thoroughly evaluate the suppression of HSPs overexpression caused by CO release from SNB in vivo, we examined HSPs levels in tumor tissue via fluorescent staining, as illustrated in Figures 4F,G, and Figure S36, S37 (Supporting Information). In the control and 2TT‐OC46B groups, there was a notable rise in HSPs levels in tumor tissues, which corresponded with increasing temperatures. Remarkably, the presence of **SNB** led to a significant inhibition of HSPs expression levels. As shown in **Figure** **5**A from the H&E staining analysis, no obvious safety margin was observed for the combined treatment strategies. Furthermore, the absence of toxicity was unequivocally confirmed by blood histopathological abnormalities or lesions, liver function tests, and hemolysis assay (Figure 5B; Figures S38 and S39, Supporting Information).

## 3 Conclusion

We have successfully synthesized a novel, small molecular photothermal agent that we have referred to as a Stealth NanoBomb (**SNB**). This agent was created through the self‐assembly process of small molecular NIR‐II AIEgens. These AIEgens were combined with a CO carrier polymer, specifically PLGA(CO), which was then coated by PEG‐lipid. This coating process ensures that the **SNB** possesses a stable and secure means of transport within the bloodstream. Our **SNB** is designed to target pancreatic cancer (PC) through the method of intravenous injection. One of the key features of the **SNB** is its ability to release CO gas in response to H_2_O_2_ which is significantly overexpressed in the tumor microenvironment, indicative of cancer‐specific targetability. In our research, we discovered that the CO gas released by the **SNB** effectively inhibits the upregulation of heat shock proteins (HSPs) in pancreatic cancer cells during photothermal therapy (PTT). This inhibition greatly improves the effectiveness of the treatment, even at mild temperatures that are below 43 °C. When compared with previous iterations of the nanobomb, the **SNB** provides a new and exciting opportunity to develop a safer and more facile system for the co‐delivery of CO gas and drugs for the treatment of diseases. This innovative approach holds significant promise for enhancing the efficacy of cancer therapy and further advancing the field of medical treatments.

## 4 Experimental Section

### Synthesis of PLGA(CO) and 2TT‐OC46B@PLGA(CO)

In a typical procedure, a mixture of Dodecacarbonyltriiron (5 mg) and PLGA‐SH (Molecular Weight ≈2000) (40 mg) was dissolved in 10 mL of tetrahydrofuran (THF) and stirred for 2 h at 60 °C under a nitrogen atmosphere. Upon completion of the reaction, the dark blue solution transitioned to a brown hue. After cooling to −20 °C, n‐hexane was introduced to afford a brown precipitate. Subsequent washing with ether and drying yielded PLGA(CO) (10.4 mg) as a brown solid, soluble in both water and chloroform. Following this, a solution comprising 7 mg of PLGA(CO) and 0.5 mg of 2TT‐OC46B in 1 mL of THF was prepared. Ultrasonication for 3 min was employed, followed by the addition of 3 mL of deionized water. THF was then removed via nitrogen blowing to induce co‐precipitation of PLGA(CO) and 2TT‐OC46B. The resulting aqueous solution underwent filtration through a 0.22 µm PVDF syringe‐driven filter (Millipore) and subsequent centrifugation for 5 min using an ultrafiltration tube (Molecular Weight ≈30 000). This washing process was repeated thrice, resulting in the acquisition of 2TT‐OC46B@PLGA(CO) NPs suitable for subsequent experimental investigations.

### Synthesis of Nanobomb2G

The lyophilized 2TT‐OC46B@PLGA(CO) powder was reconstituted in deionized water to yield a concentration of 5 mg mL^−1^. Separately, DSPE‐mPEG2K (2 mg) was dissolved in 1 mL of dichloromethane. Upon mixing the solutions, ultrasonication was performed for 5 min, followed by removal of the dichloromethane under a stream of nitrogen to facilitate co‐precipitation of 2TT‐OC46B@PLGA(CO) and DSPE‐mPEG2K. The resulting aqueous solution underwent filtration through a 0.22 µm PVDF syringe‐driven filter, followed by triple washing utilizing a 100 K centrifugal filter unit under centrifugation at 5000 rpm for 5 min each.

### Photothermal Activity and Photothermal Conversion Efficiency

A solution of Nanobomb2G at varying concentrations (0.4, 0.8, 1.2, 1.6, and 2.0 mg mL^−1^) was subjected to irradiation with an 808 nm laser at 2.5 W cm^−2^ for a duration of 3 min. Thermal changes were monitored using a thermal imaging camera. Subsequently, the photothermal performance of the aqueous Nanobomb2G solution (2.0 mg mL^−1^) was evaluated under different power densities of 808 nm laser irradiation (0.5, 1.0, 1.5, 2.0, and 2.5 W cm^−2^) using the same methodology. To investigate the photothermal conversion efficiency, the Nanobomb2G solution (2.0 mg mL^−1^, 150 µL) was exposed to continuous irradiation from an 808 nm laser (2.5 W) for 300 s, followed by cessation of the NIR laser. Simultaneously, an infrared thermal camera was employed to monitor both the temperature elevation and subsequent cooling. The photothermal conversion efficiency (η) of Nanobomb2G was determined to be 33.1%.

### Detect CO Release In Vitro by HEMOGLOBIN

Hemoglobin (Hb) release of CO in PBS was quantified spectrophotometrically through the conversion of hemoglobin to carboxyhemoglobin (HbCO). Initially, hemoglobin sourced from bovine erythrocytes (Energy Chemicals) at a final concentration of 5.5 µm was fully dissolved in phosphate‐buffered saline (PBS) at pH 7.4 (10 mm). Subsequently, under a nitrogen atmosphere, sodium dithionite (SDT, 1.5 mg) was added to reduce the hemoglobin. Following this, an aqueous solution of Nanobomb2G (100 mg mL^−1^) (50 µL) underwent deoxygenation by nitrogen gas bubbling before being introduced into the Hb solution. The entire reaction mixture (4 mL) was promptly sealed within a 4 mL UV quartz cuvette. UV adsorption spectra of the solution within the range of 400–450 nm were recorded using a UV–vis spectrophotometer. To enhance precision and mitigate confounding factors, the conversion of Hb to HbCO was quantified utilizing two robust absorption bands at 410 and 430 nm, corresponding to HbCO and Hb, respectively. Utilizing the Beer–Lambert law, the percentage of Hb‐to‐HbCO conversion (x) and the concentration of released CO, coordinated with Hb, were calculated accordingly.

(1) $$C_{CO}=C_{Hb}\;.\;x=\frac{528.6\times I_{410nm}-304\times I_{430nm}}{216.5\times I_{410nm}+442.4\times I_{430nm}}C_{Hb}$$

### Confocal Fluorescence Analysis of CO Release in Cells

Panc02 cells were subjected to confocal fluorescence analysis to evaluate the efficacy of Nanobomb2G. Cells were cultured in eight‐well plates at a density of 12 000 cells per well, with 150 µL of DMEM medium. Four experimental groups were established: PBS, 2TT‐OC46B, PLGA(CO), and Nanobomb2G, each supplemented with 60 µg mL^−1^. Following a 40 min incubation period, a CO probe at a concentration of 40 µg mL^−1^ was introduced to each well and incubated for an additional 20 min. Subsequently, the residual culture medium was removed by washing with PBS, and the fluorescence signal within the range of 560–600 nm was recorded using a confocal microscope.

### Western Blot Assays

To assess the expression levels of HSP70, Panc02 cells were seeded into 12‐well culture plates at a density of 1 × 10^5^ cells per well, with 1 mL of DMEM medium. The experiment comprised seven distinct groups: 1) PBS + NIR; 2) heat incubation at 43 °C for 10 min; 3) PBS alone; 4) 2TT‐OC46B treatment; 5) 2TT‐OC46B + NIR exposure for 5 min; 6) PLGA(CO) treatment; 7) PLGA(CO) + NIR exposure for 5 min; 8) Nanobomb2G treatment; 9) Nanobomb2G + NIR exposure for 5 min. Subsequently, cells were harvested using trypsin and lysed in lysis buffer. Protein content was determined using a BCA protein assay. The proteins were then separated by sodium dodecyl sulfate‐polyacrylamide gel electrophoresis (SDS‐PAGE) and transferred to a polyvinylidene fluoride (PVDF) membrane. Following a 1 h blocking step with 5% dried skimmed milk, the membrane was incubated overnight with the appropriate primary antibody on a shaker (at 4 °C), followed by incubation with the secondary antibody for 1 h at room temperature. Finally, the membrane was visualized using an ECL plus detection system.

### In Vivo Anti‐Tumor Effect

4T1 tumor‐bearing C57 mice were divided into eight groups, with three mice in each group: 1) PBS; 2) PBS + NIR exposure at 1.5 W for 5 min; 3) PLGA(CO)@PEG treatment; 4) PLGA(CO)@PEG + NIR exposure; 5) 2TT‐OC46B@PEG treatment; 6) 2TT‐OC46B@PEG + NIR exposure; 7) Nanobomb2G treatment; 8) Nanobomb2G + NIR exposure. These solutions were administered intravenously to the mice. After 12 h, the tumor region was subjected to irradiation with 808 nm NIR, ensuring that the tumor temperature did not exceed 45 °C, monitored using a photothermal camera. Tumor volumes and mouse weights were measured every 2 days. On day 14, the mice were euthanized, and major organs and tumors were collected for histological examination.

## Conflict of Interest

The authors declare no conflict of interest.

## Author Contributions

G. M., Q. D., Y.W., and Z.Z. contributed equally to this work. All authors have given approval to the final version of the manuscript.

## Supporting information



Supporting Information

## Data Availability

The data that support the findings of this study are available from the corresponding author upon reasonable request.
