# Supplementary material for: Precision Photothermal Therapy at Mild Temperature: NIR‐II Imaging‐Guided, H2O2‐Responsive Stealth Nanobomb
Source: Adv Healthc Mater. 2024 Oct 10;14(26):2402767. doi: 10.1002/adhm.202402767 (PMC12506850; doi:10.1002/adhm.202402767)
Supplement: Supplementary file 1 — Supporting Information [file ADHM-14-0-s001.docx]

Supporting Information

Precision Photothermal Therapy at Mild Temperature: NIR-II Imaging-Guided, H_2_O_2_-Responsive Stealth Nanobomb

Gongcheng Ma,^1^^[‡]^ Qihang Ding,^3[‡]^ Yue Wang,^2[‡]^ Zhiwei Zhang,^2,4[‡]^ Yuding Zhang,^2^ Hui Shi,^5^ Lintao Cai,^2,6^ Ping Gong,^2^* Pengfei Zhang,^2^* Zhen Cheng^5^* and Jong Seung Kim^3^*

1. School of Life Science and Technology, Xinxiang Medical University, Xinxiang, 453003 China

2. Guangdong Key Laboratory of Nanomedicine, CAS-HK Joint Lab of Biomaterials, CAS Key Laboratory of Biomedical Imaging Science and System, Shenzhen Engineering Laboratory of Nanomedicine and Nanoformulations, CAS Key Lab for Health Informatics, Shenzhen Institutes of Advanced Technology, Chinese Academy of Sciences, Shenzhen 518055, P. R. China. E-mail: pf.zhang@siat.ac.cn

3. Department of Chemistry, Korea University, Seoul 02841, Korea. E-mail: jongskim@korea.ac.kr

4. University of Chinese Academy of Sciences, Beijing, 100049, P.R. China.

5. State Key Laboratory of Drug Research, Molecular Imaging Center, Shanghai Institute of Materia Medica, Chinese Academy of Sciences, Shanghai 201203, China; E-mail: zcheng@simm.ac.cn

6. Sino-Euro Center of Biomedicine and Health, Luohu, Shenzhen 518024, China

* Correspondence author.

[‡] These authors contributed equally.

**Experimental details:**

**Characterization.** The UV-vis absorption spectra were obtained from UV-8000 spectrophotometer (INESA). The emission spectra were obtained from Edinburgh FS5 (Ediburgh Instruments). Fourier-transform infrared spectra (FT-IR) were obtained by a Vertex Perkin-Elmer 580BIR spectrophotometer (Bruker). Dynamic light scattering (DLS) and Zeta potential were obtained by using a Malvern instrument Zetasizer Nano system. Thermal images were recorded using a FLIR T420 thermal camera.

**Cell culture.** Panc-02and LO2 cell were seeded in DMEM medium supplemented with 10% FBS, penicillin (100 units mL^-1^) and streptomycin (100 μg mL^-1^) in 5% CO_2_ at 37°C.

**CCK-8 assay.** The biocompatibility assay of Stealth nanobomb was conducted on Panc02 and LO2 cells respectively. The cells were cultivated in a 96-well plate (8000 cells per well, 100 μL of DMEM medium). Add continuous concentrations (0, 1, 2, 3, 4, 5, 6, 7, 8, 9 and 10 μg/mL) Stealth nanobomb to the above medium. After 24 h, the incubated cells were irradiated with 808 nm laser (5 W/cm^2^, 5 min). Monitored by infrared camera, the maximum temperature during irradiation is 43 ℃. Continue to incubate for 24 h, add CCK-8 reagent, and then detect with microplate reader after 1.5 h. The experiment was divided into four groups: (1) Panc02; (2) Panc02 + NIR; (3) LO2; (4) LO2 + NIR.

**Animal model.** Female C57 mice (six weeks-old) were purchased from the gempharmatech, Guangzhou, China. The Panc02 tumor model was established by hypodermic injection with Panc02 cells into C57 mice. When the tumor volume is about 100 mm^3^, the in vivo experiments were carried out. All animal experiments were performed under the protocols approved by the Animal Care and Use Committee (Shenzhen Institutes of Advanced Technology, Chinese Academy of Sciences) (Serial number: Serial number: SIAT-IACUC-210701-YYS-GP-A1974).

**Supplementaty Figures:**


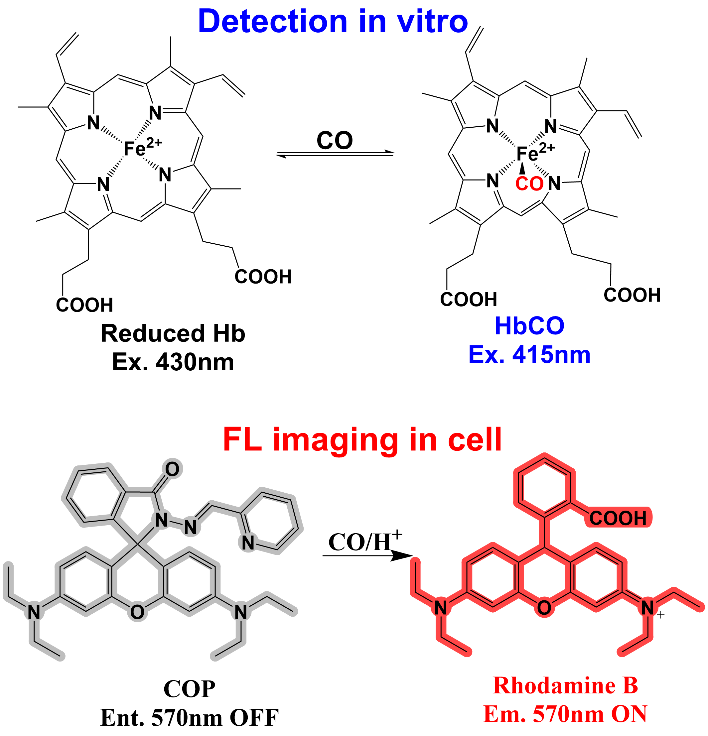


**Scheme S1.** Detection of CO release of Stealth nanobomb in vitro and cell experiments.


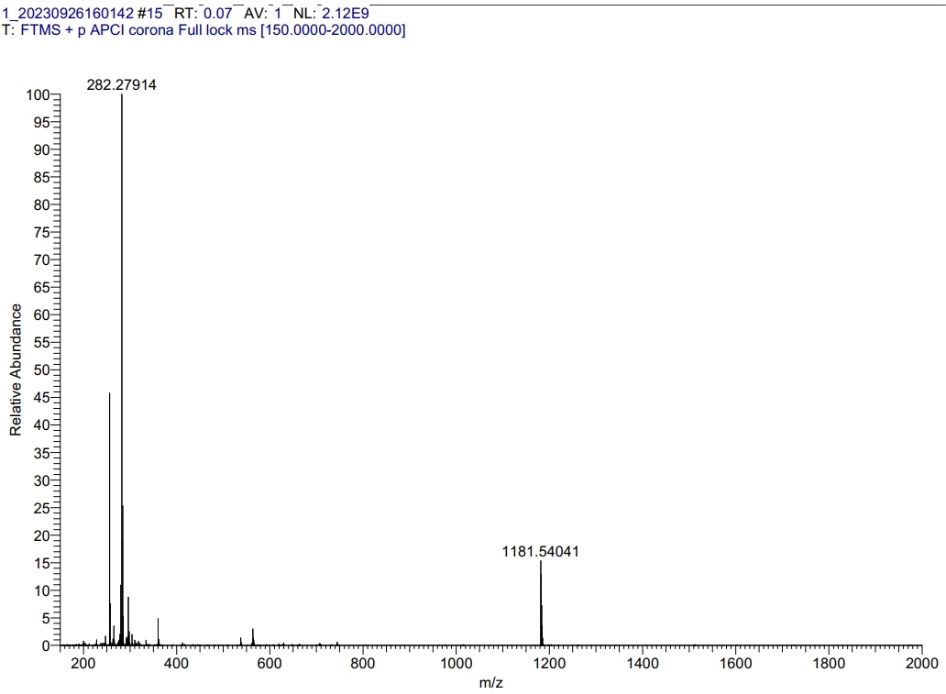


**Figure S1.** The ESI-Mass spectrum of 2TT-OC46B. RMS (ESI) calcd. for C_74_H_80_N_6_S_4_ [M + H]^+^= 1181.54041.


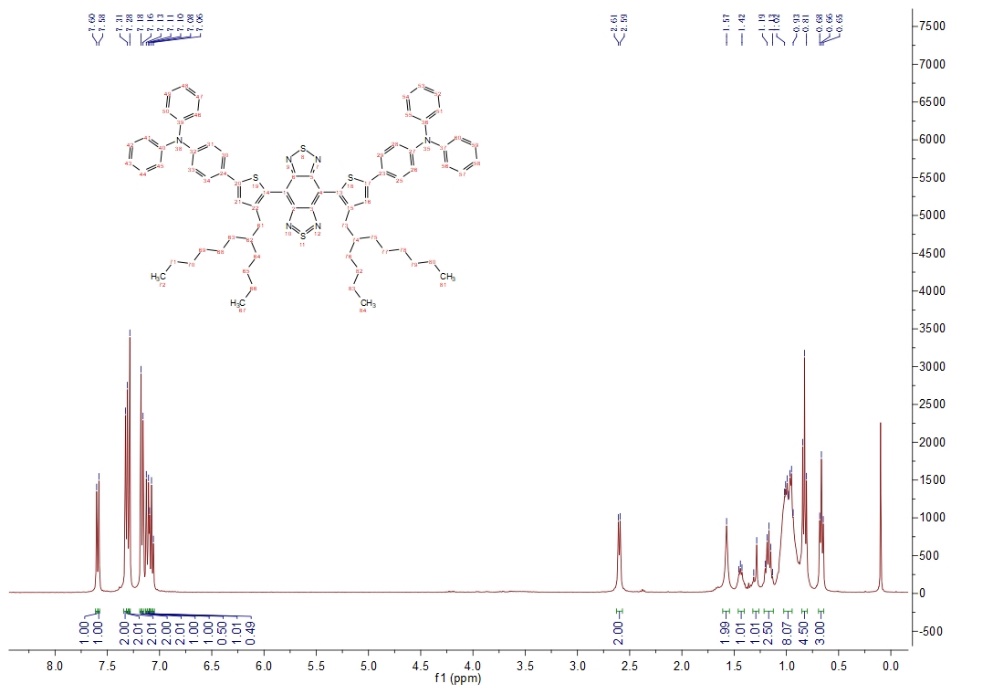


**Figure S2.** The HNMR spectrum of 2TT-OC46B. ^1^H NMR (400 MHz, CDCl_3_) δ = 7.60 (s, 2H), 7.58 (s, 2H), 7.33 (s, 4H), 7.31 (s, 4H), 7.28 (s, 5H), 7.18 (s, 5H), 7.16 (s, 4H), 7.13 (s, 2H), 7.11 (s, 2H), 7.10 (s, 1H), 7.08 (s, 2H), 7.06 (s, 1H), 2.60 (d, *J*=6.9, 2H), 1.57 (s, 2H), 1.46 – 1.40 (m, 1H), 1.30 (d, *J*=11.4, 1H), 1.17 (dt, *J*=14.1, 7.0, 2H), 1.02-0.94 (m, 8H), 0.83 (t, *J*=7.3, 4H), 0.66 (t, *J*=6.7, 3H).


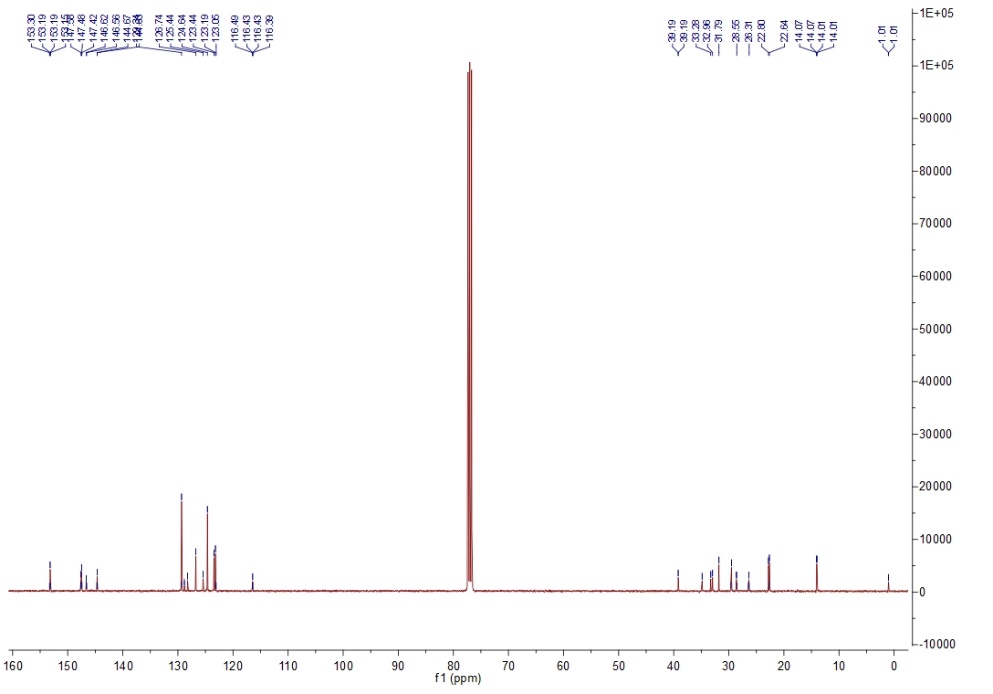


**Figure S3.** The CNMR spectrum of 2TT-OC46B. ^13^C NMR (101 MHz, CDCl_3_) δ = 153.30, 153.19, 153.19, 153.15, 147.66, 147.58, 147.58, 147.54, 147.48, 147.48, 147.42, 146.67, 146.62, 146.62, 146.56, 144.72, 144.67, 144.67, 144.63, 129.42, 129.34, 129.34, 128.83, 128.80, 128.25, 128.25, 126.74, 126.74, 125.44, 125.44, 124.64, 124.64, 123.44, 123.44, 123.19, 123.19, 123.11, 123.05, 116.49, 116.43, 116.43, 116.39, 39.19, 39.19, 34.85, 34.85, 33.28, 33.28, 32.96, 32.96, 31.79, 31.79, 29.59, 29.51, 29.51, 28.63, 28.63, 28.60, 28.55, 26.43, 26.36, 26.36, 26.31, 22.80, 22.80, 22.71, 22.64, 22.64, 14.07, 14.07, 14.01, 14.01, 1.01, 1.01.


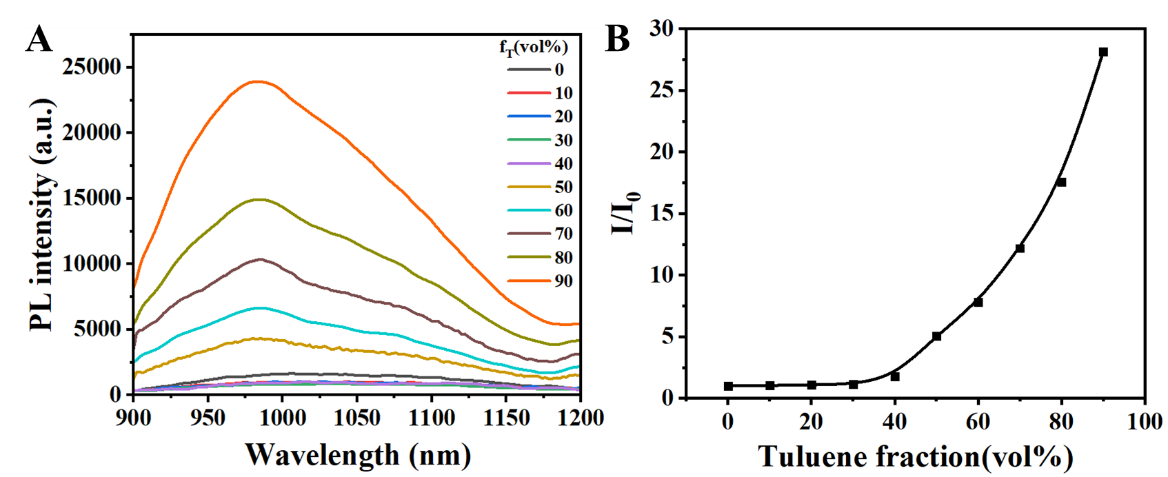


**Figure S4.** The AIE property of 2TT-OC46B. (A) Photoluminescence (PL) spectra of 2TT-OC46B in pure THF and its PL spectra in a THF/water mixtures with different water fractions (*f*_w_s). (B) Plot of the maximum PL intensities of 2TT-OC46B in pure THF and THF/water mixture.


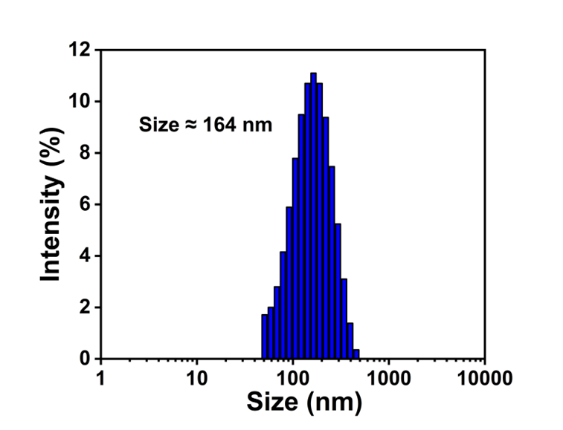


**Figure S5.** The particle size of 2TT-OC46B@PLGA(CO) measured by DLS.


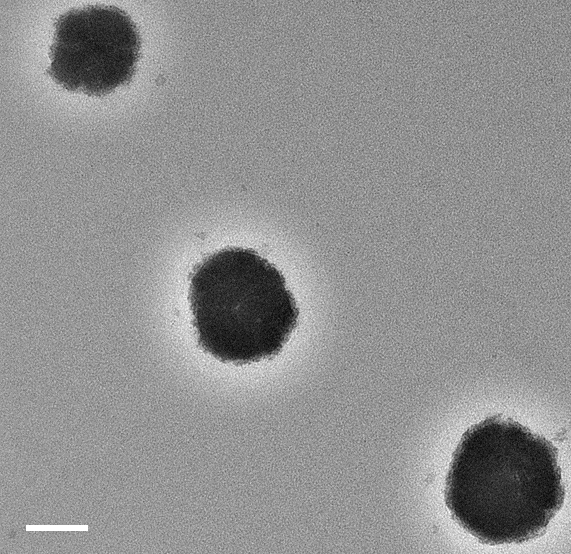


**Figure S6.** The TEM image of 2TT-OC46B@PLGA(CO). Scale bar 100 nm.


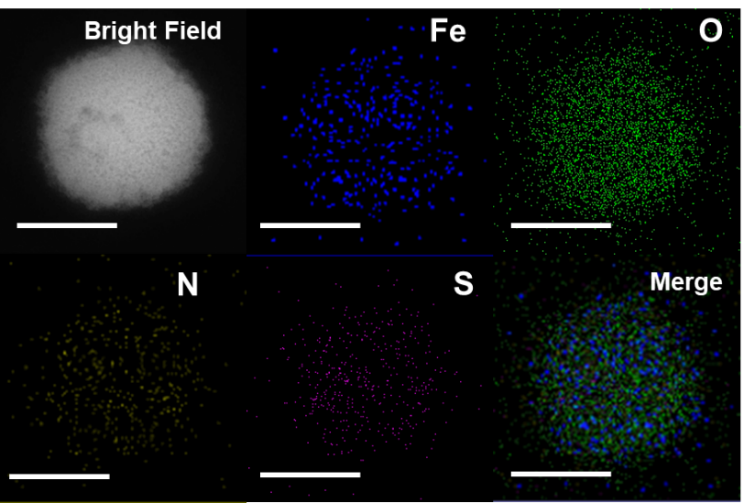


**Figure S7.** The EDS elemental mapping of 2TT-OC46B@PLGA(CO). Scale bar 100 nm

**Figure S8.** The EDS elemental mapping of Stealth nanobomb. Scale bar 200 nm





**Figure S9.** The surface zeta potential of 2TT-OC46B@PLGA(CO) and Stealth nanobomb.


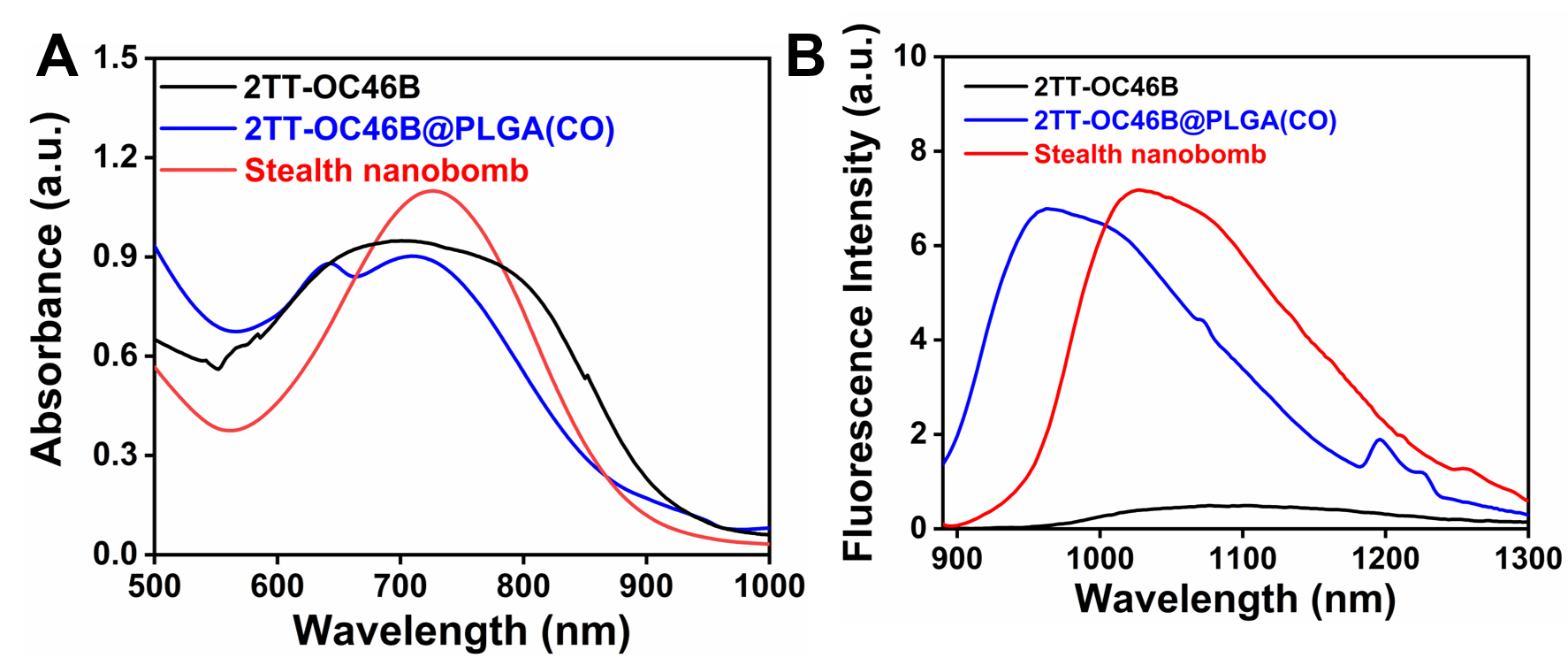


**Figure S10.** The absorbance spectra (A) and fluorescence spectra of (B) of 2TT-OC46B, 2TT-OC46B@PLGA(CO) and Stealth nanobomb.


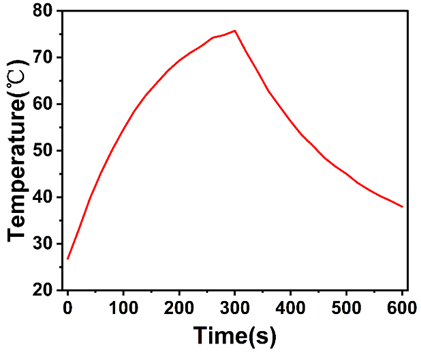


**Figure S11.** The photothermal conversion efficiency (PCE) about 33.1% of the as-proposed Stealth nanobomb (100 mg/mL, 100 mL) was calculated using 5 W/cm^2^ of an 808 nm laser.


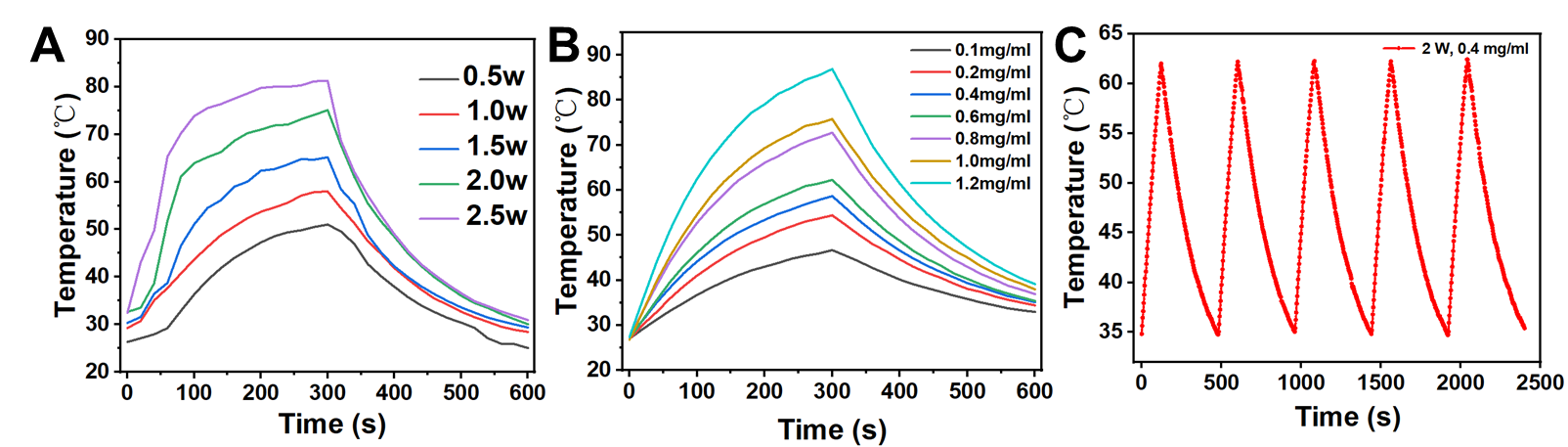


**Figure S12.** The temperature changes of Stealth nanobomb solution with the different of (A) laser power and (B) concentrations; (C) The temperature changes of Stealth nanobomb in six cycles under 2 W laser irradiation.


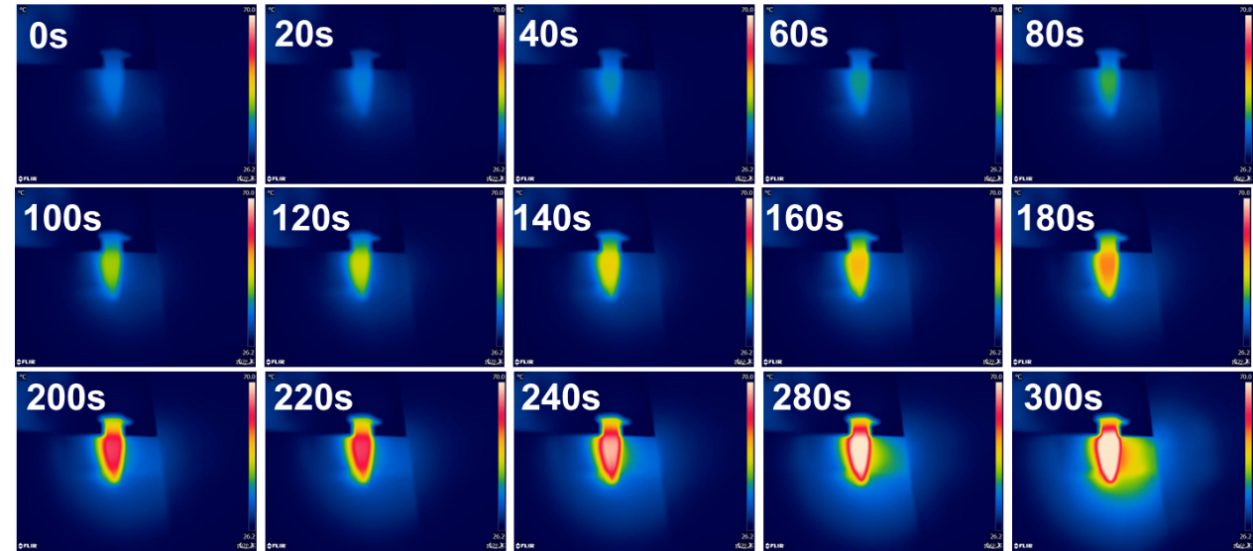


**Figure S13.** The thermal images during photothermal conversion process.





**Figure S14.** The temperature changes of Stealth nanobomb and ICG nanoparticle in six cycles under 2.5 W laser irradiation.





**Figure S15.** Changes in UV absorption of nanobomb^2G^ in DMEM over time.


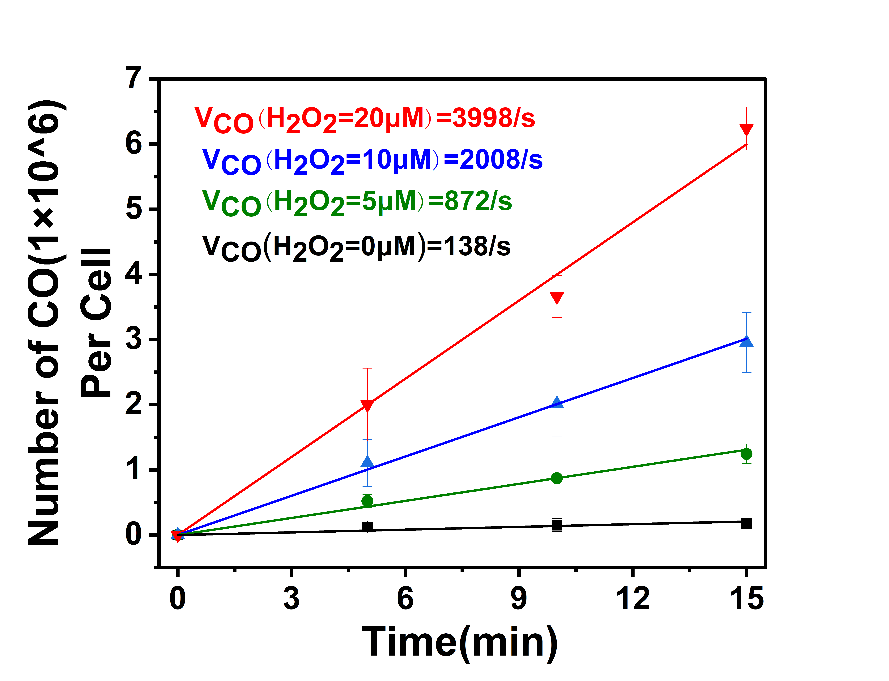


**Figure S16.** The number of CO (x10^6^) released by Stealth nanobomb per cancer cell was calculated with time.


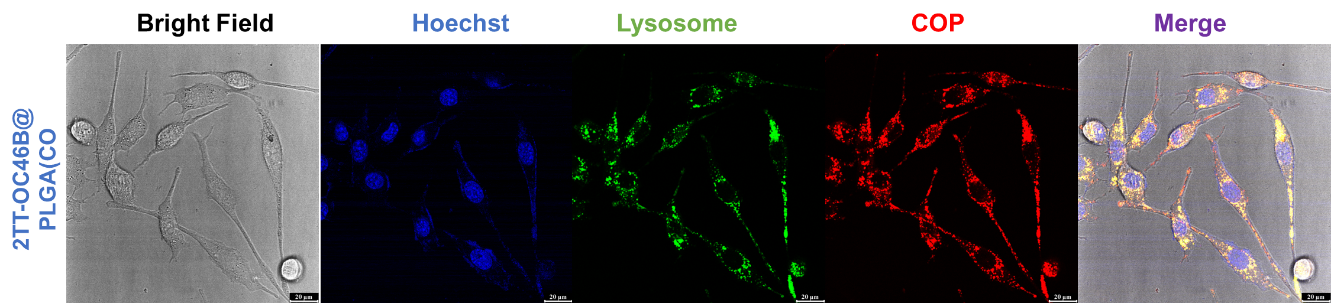


**Figure S17.** The expression of CO in Panc02 cells treated with 2TT-OC46B@PLGA(CO) imaged by the confocal fluorescence microscope.





**Figure S18.** The fluorescence intensity of CO probe in the presence of Stealth nanobomb.


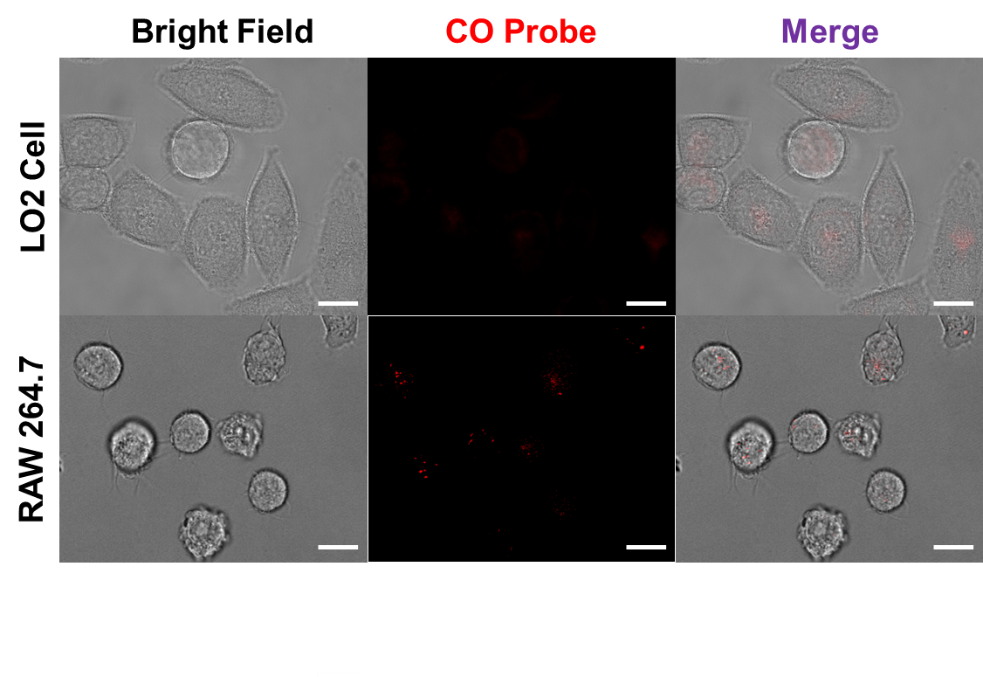


**Figure S19.** The expression of CO in LO2 and RAW264.7 cells were imaged by the confocal fluorescence microscope. (Scale bar 10 μm)


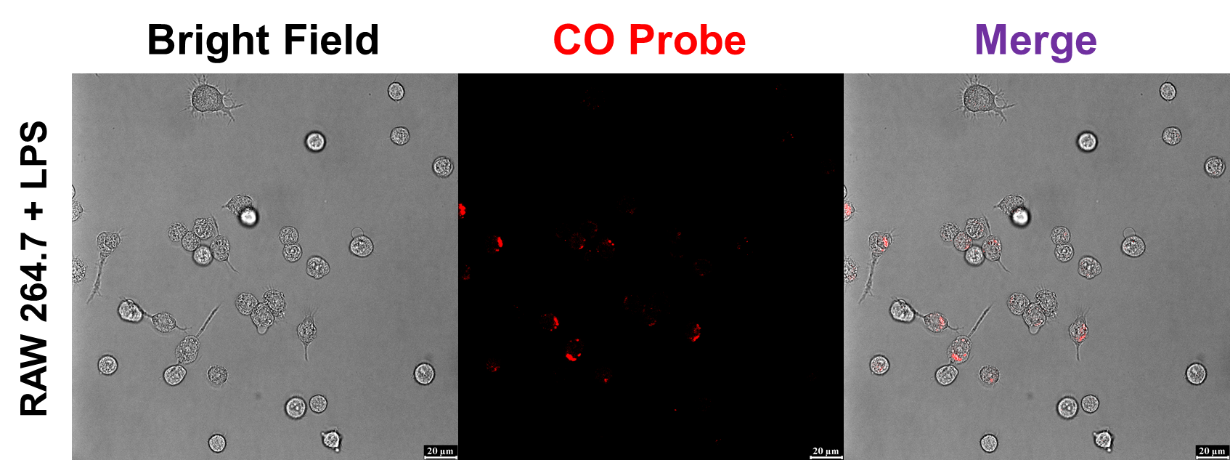


**Figure S20.** Confocal fluorescence imaging of CO release during LPS induced cell damage or oxidative stress.


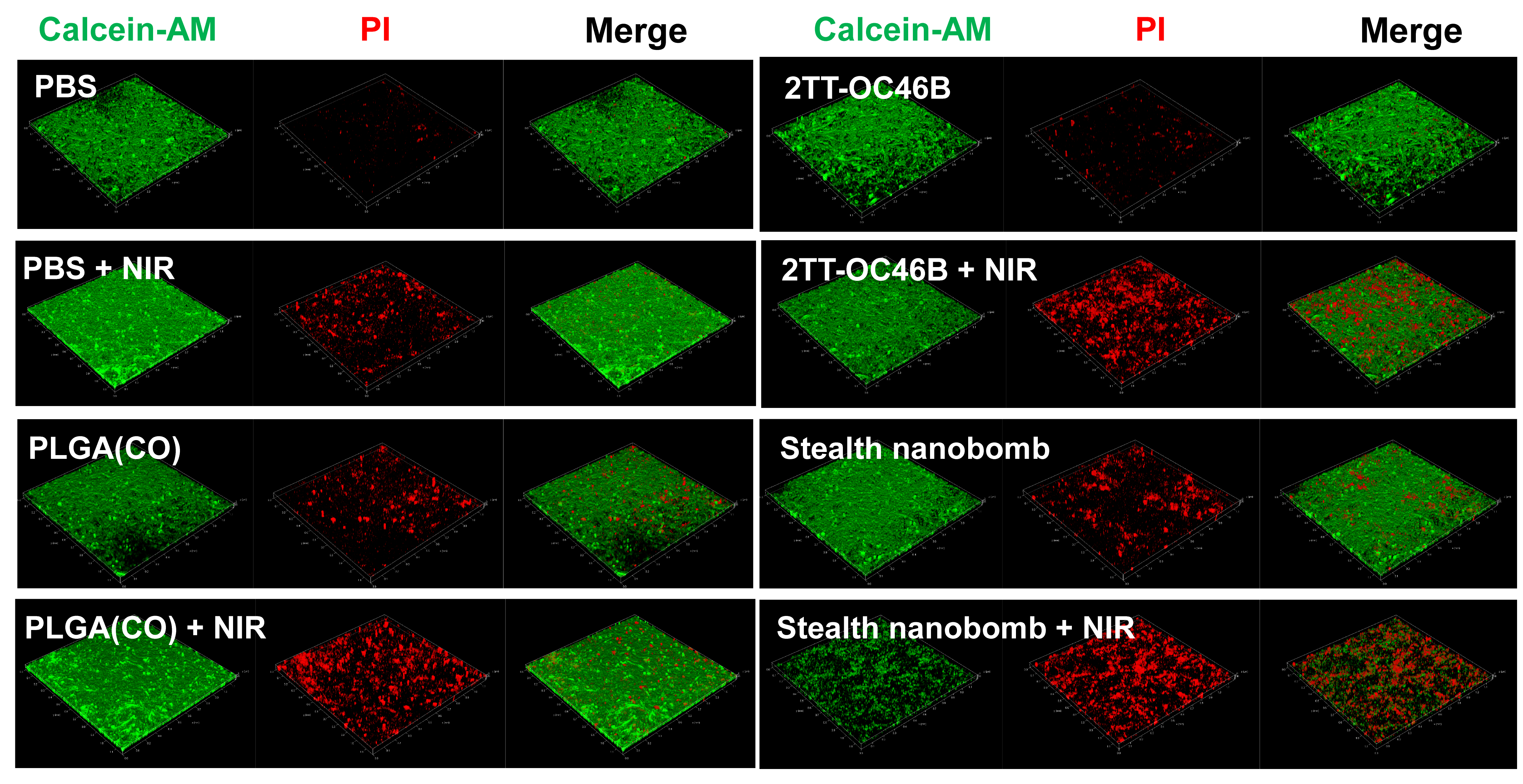


**Figure S21.** The survival of Panc02 cells in the dead and alive staining experiment measured by 3D confocal fluorescence microscope.


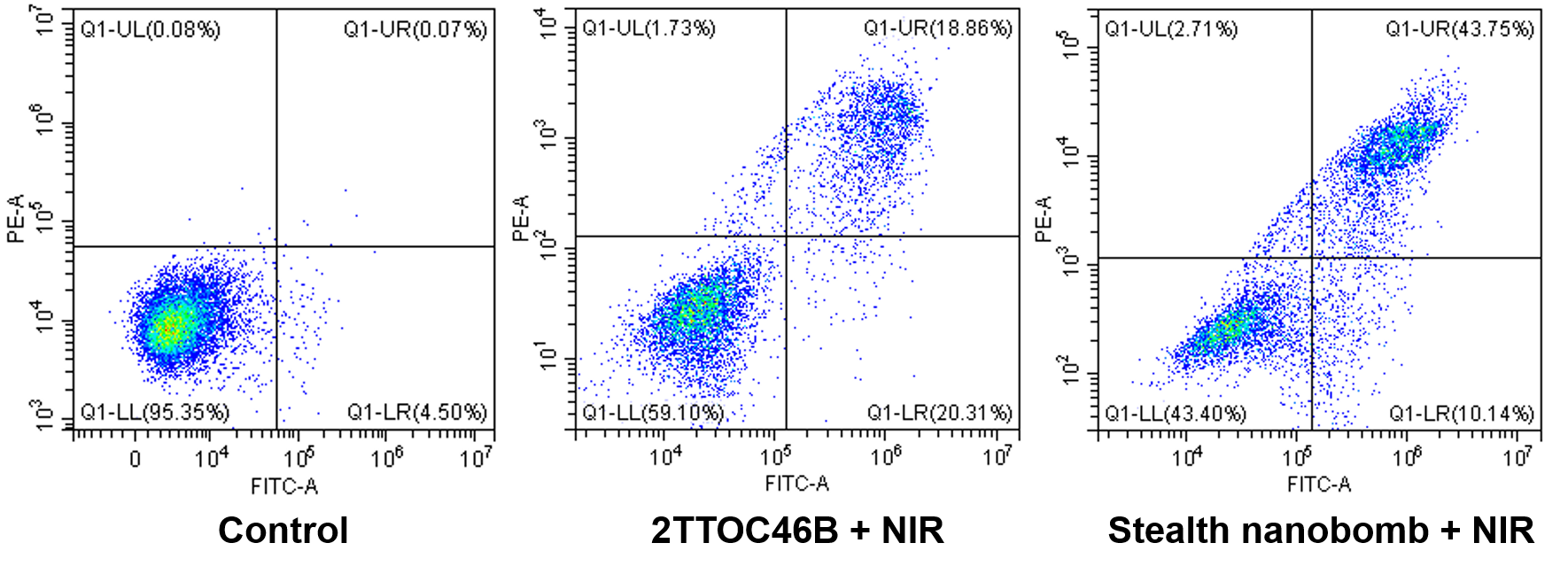


**Figure S22**. Representative flow cytometry plots and quantitative analysis showing the apoptosis experiment results of Panc02 cells.


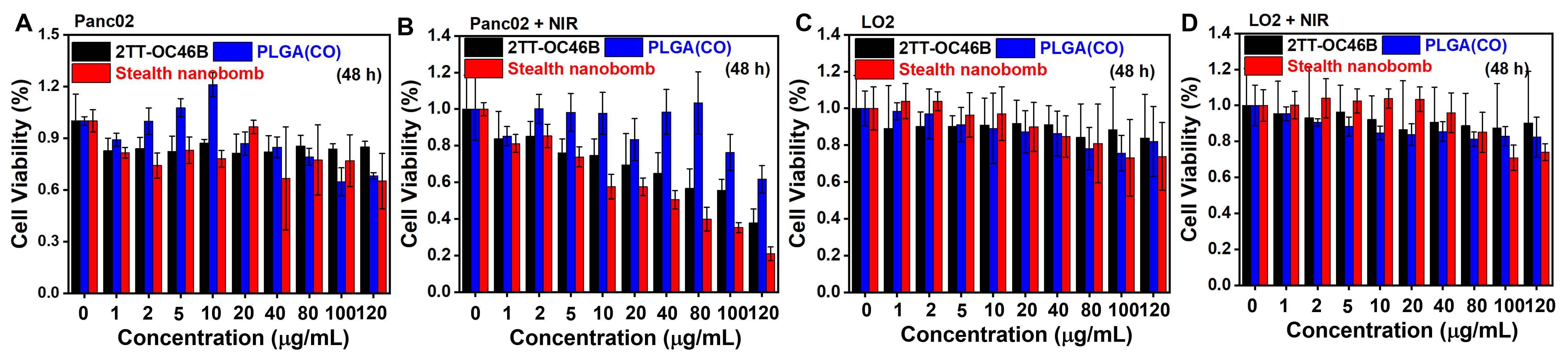


**Figure S23.** Cell viability of Panc02 and LO2 cells incubated with Stealth nanobomb under 808 nm NIR in 48 h.


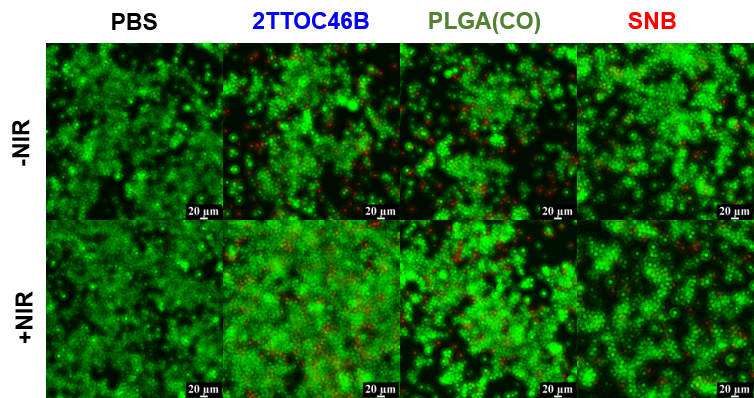


**Figure S24.** The viability of RAW264.7 cells was assessed using confocal fluorescence microscope in the live/dead staining experiment. (Scale bar 20 μm)


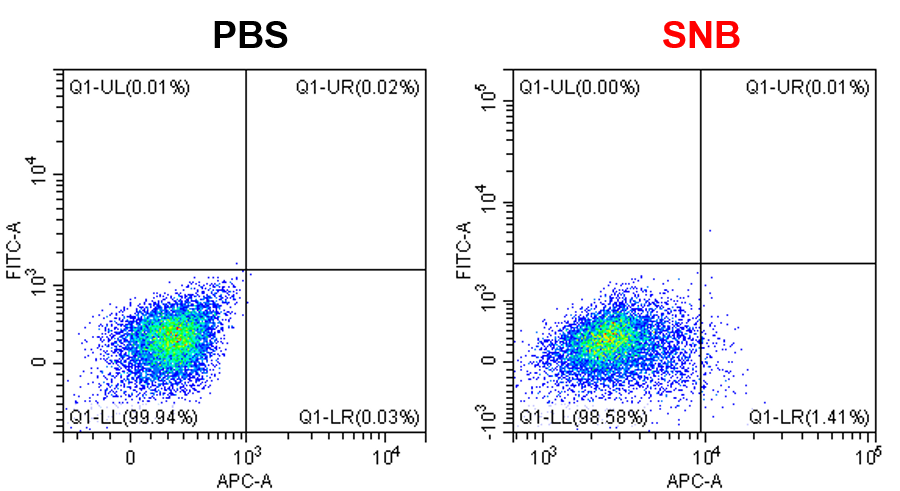


**Figure S25.** The representative flow cytometry plots and quantitative analysis showing SNB does not induce macrophage polarization.





**Figure S26.** Corresponding gray analysis of HSP70 with different treatments in Panc02 cells.


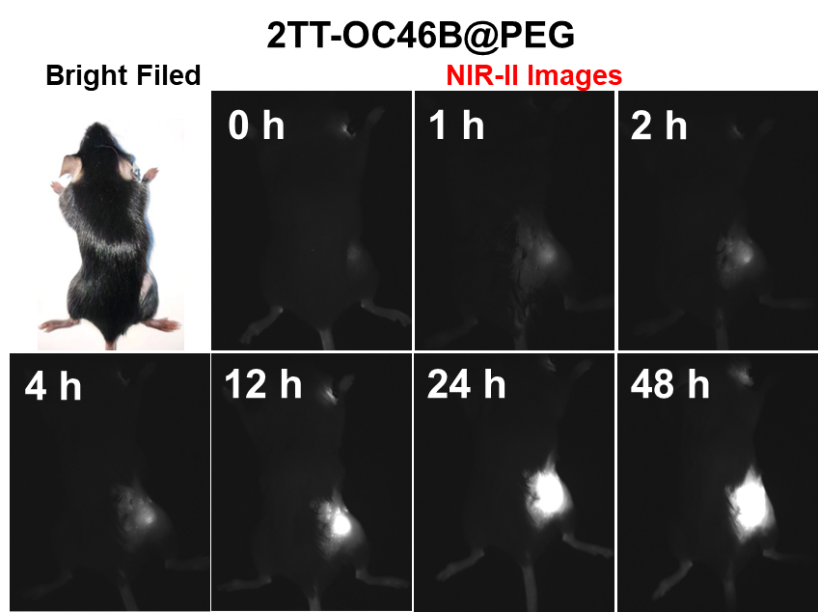


**Figure S27.** The NIR-II fluorescence images of panc02 tumor-bearing mice after intravenous injection of 2TT-OC46B@PEG.





**Figure S28.** The fluorescence intensity curve of tumor within 48 h after intravenous injection of 2TT-OC46B@PEG and Stealth nanobomb (SNB).





**Figure S29.** Pharmacokinetic profiles of the Stealth nanobomb for 3 days post injection. Total fluorescence intensity at 1 h for each animal was used as 100% to normalize the plasma concentrations of the Stealth nanobomb at different times.


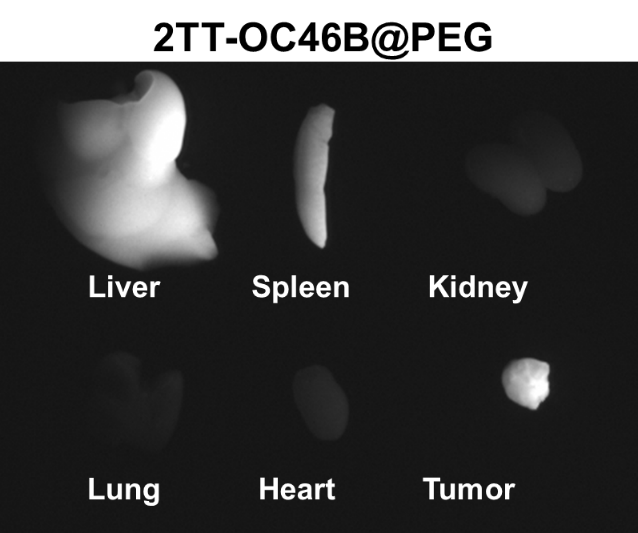


**Figure S30.** The NIR-II images of heart, liver, spleen, lung, kidney, and tumor of mice after intravenous injection of 2TT-OC46B@PEG


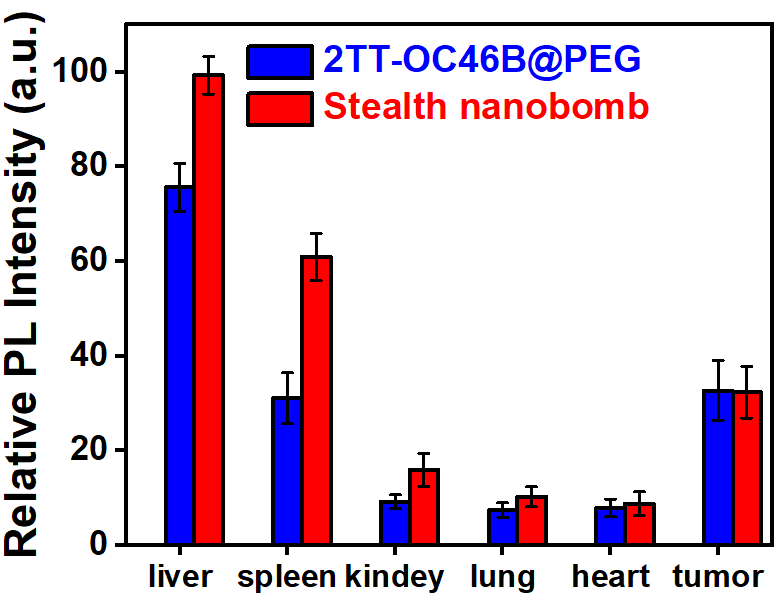


**Figure S31.** The relative fluorescence intensity curve of heart, liver, spleen, lung, kidney, and tumor.


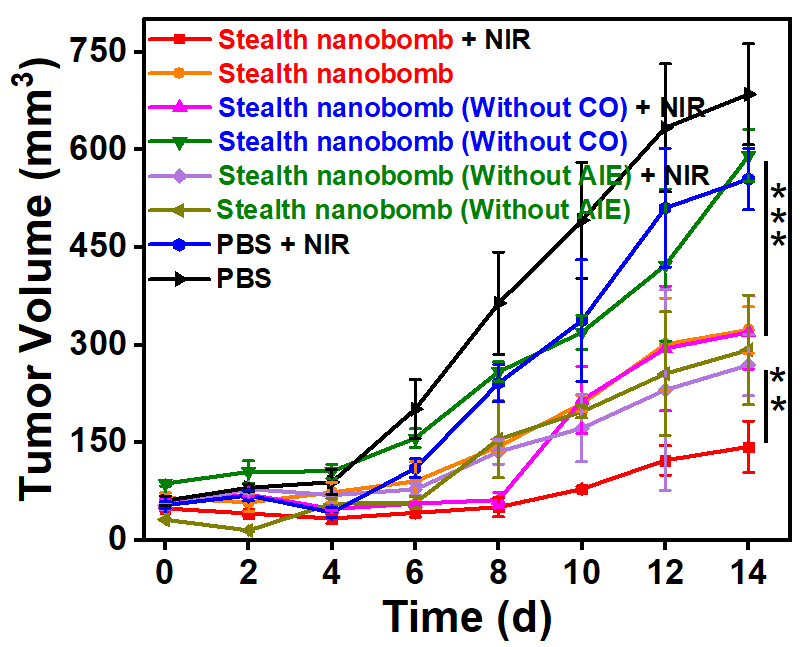


**Figure S32.** Change curve of tumor volume in mice within 14 days.


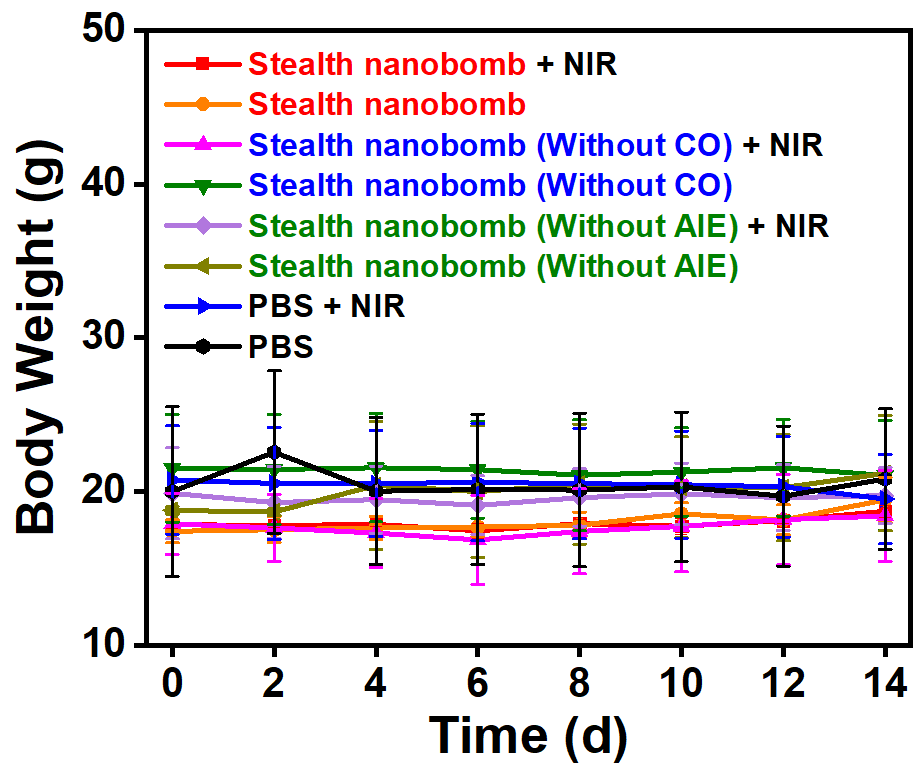


**Figure S33.** Weight changes of mice during treatment.


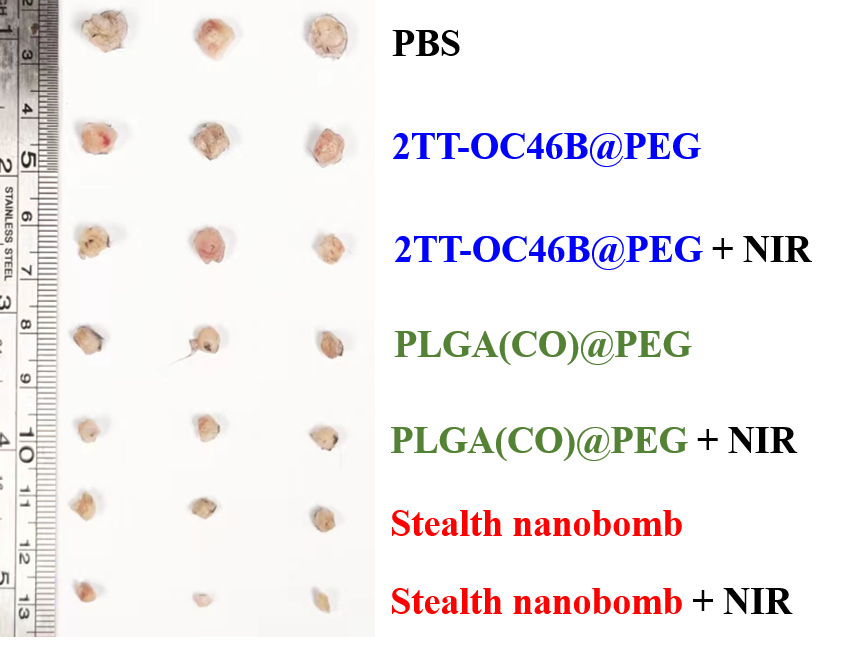


**Figure S34.** Picture of separated tumor after treatment.


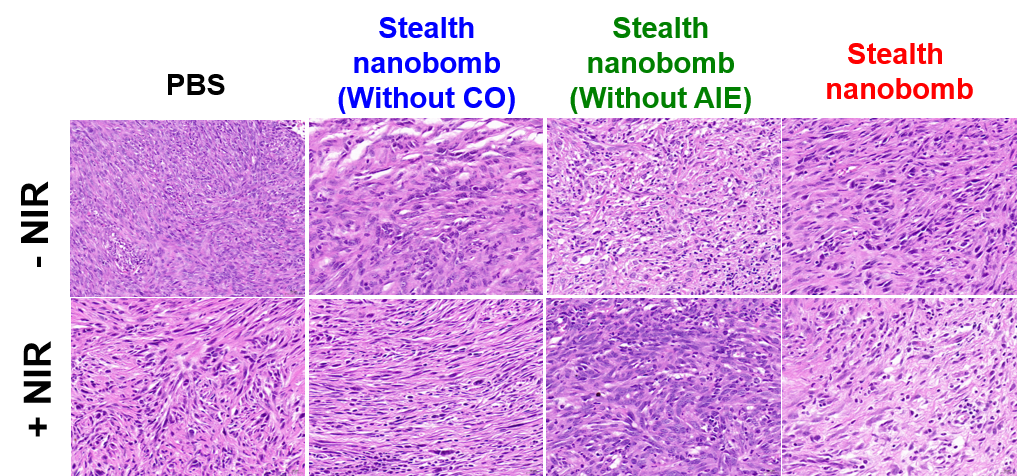


**Figure S35.** The histological analysis of treated tumors by the H&E staining. Scale bar 50 μm.


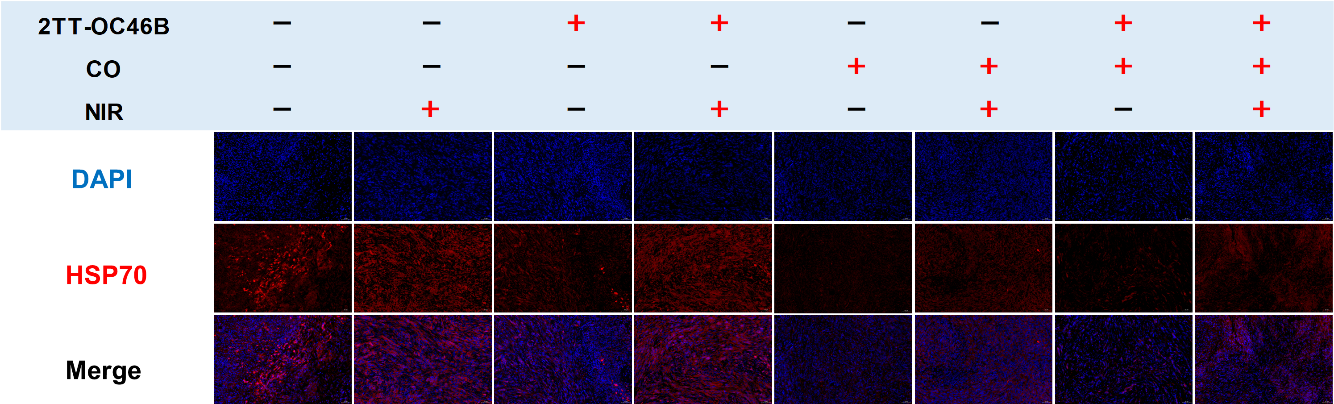


**Figure S36.** Staining sections of tumor tissues showed the expression of HSP70 under different treatments. Scale bar 100 μm.


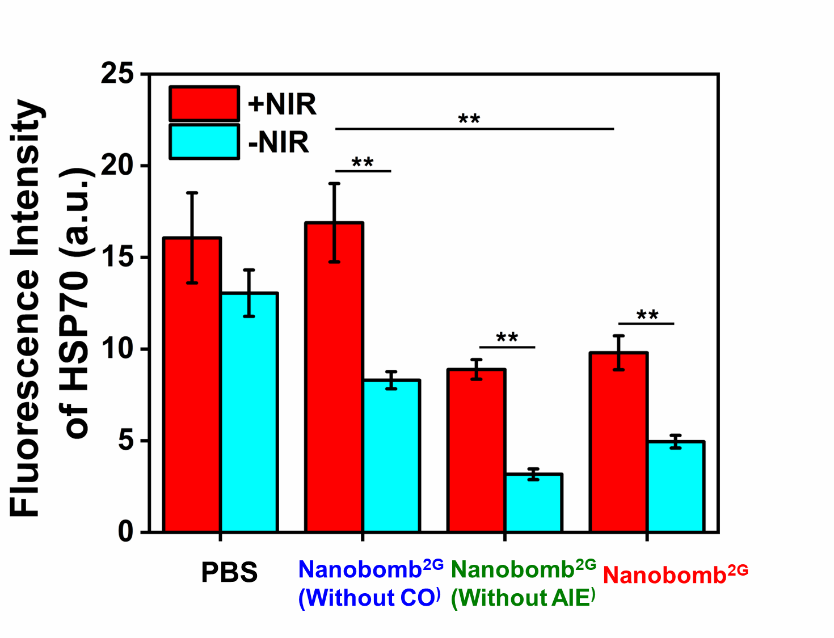


**Figure S37.** Fluorescence intensity showed significant differences under different conditions. (*P<0.05, **P<0.005, and ***P<0.0005).


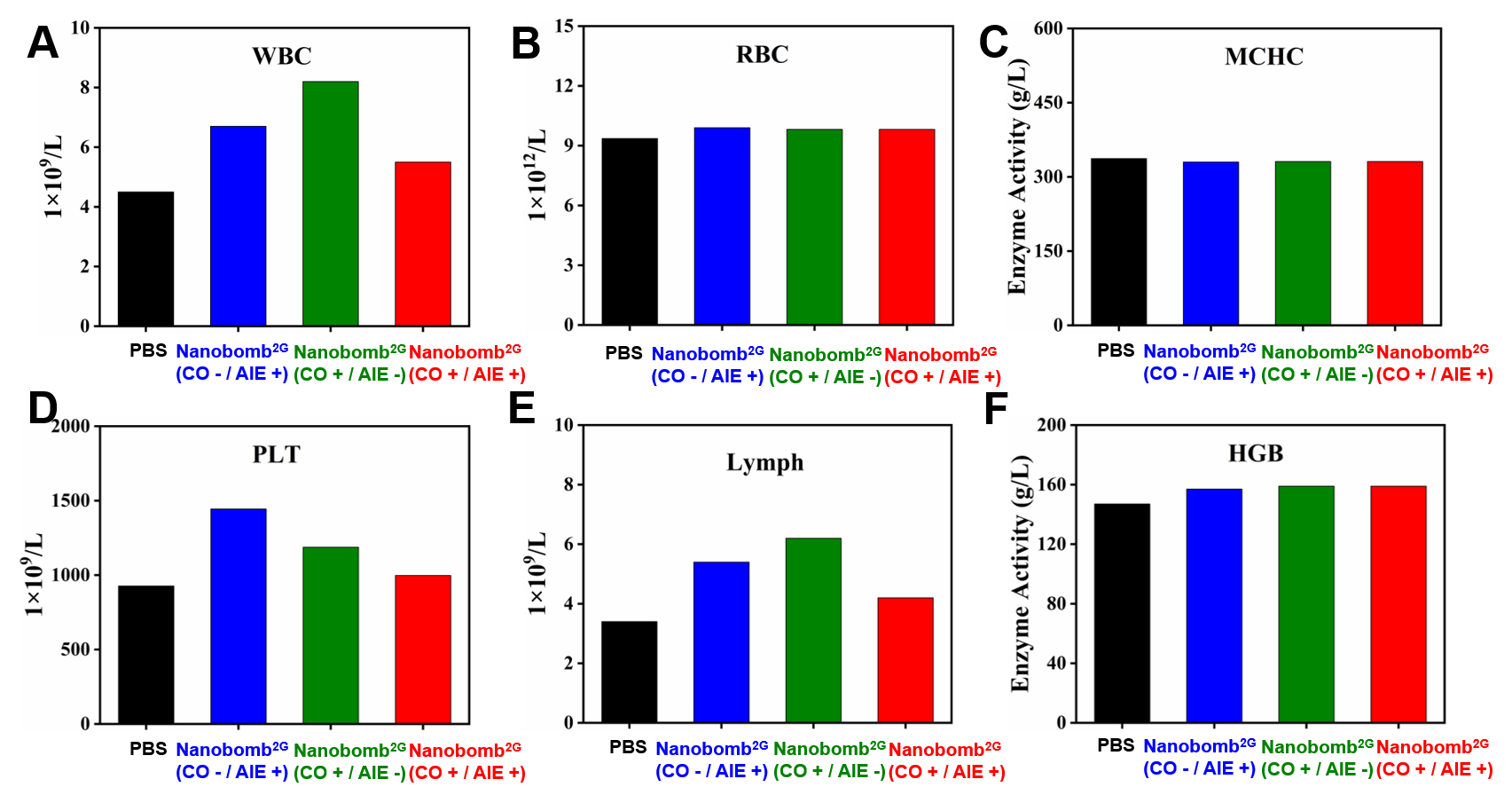


**Figure S38.** Blood analysis of mice treated with Stealth nanobomb.


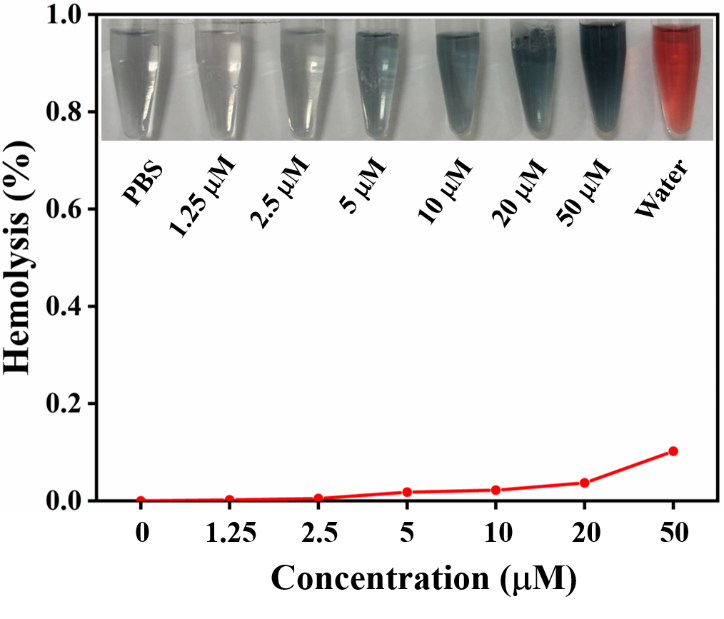


**Figure S39.** Hemolysis experiments of Stealth nanobomb with different concentrations.
